# Personal

**Published:** 1889-04

**Authors:** 


### Personal.

DR. CHAS. A. RING, who has for the past eight years been physician to the Erie County Insane Asylum, has resigned, and resumed his practice in this city, at 364 Niagara street.

DR. WM. H. PARKER, of this city, has been appointed to fill the vacancy occasioned by Dr. Ring's resignation, and has already entered upon the duties thereof.

